# Treating early-stage centrally-located non-small cell lung cancer with DCAT-SBRT in centers lacking the VMAT technique: a comprehensive study

**DOI:** 10.3389/fonc.2024.1431082

**Published:** 2024-12-05

**Authors:** Yangyang Huang, Jun Yang, Rui Song, Tingting Qin, Menglin Yang, Yibao Liu

**Affiliations:** ^1^ School of Nuclear Science and Engineering, East China University of Technology, Nanchang, Jiangxi, China; ^2^ Department of Radiotherapy, the Second Affiliated Hospital of Zhengzhou University, Zhengzhou, Henan, China

**Keywords:** DCAT, SBRT, inoperable early-stage centrally-located NSCLC, dosimetric parameter, plan complexity, delivery time, γ-passing rate, interplay effect

## Abstract

**Background:**

Volumetric-modulated arc therapy (VMAT) may have the highest overall performance for stereotactic body radiotherapy (SBRT) treatment of inoperable early-stage NSCLC. However, in centers lacking the VMAT technique, the dynamic conformal arc therapy (DCAT) technique is potentially the best option for small and rounded NSCLC-SBRT. Therefore, we will comprehensively analyze the advantages of the DCAT versus the other techniques except VMAT in terms of dosimetry, plan complexity, delivery time, γ-passing rates and the interplay effect.

**Methods:**

36 patients with early-stage centrally located NSCLC with PTV volumes < 65 cc were enrolled. All patients were redesigned with 50Gy/5f, and 100% of the prescribed dose was normalized to cover 95% of the PTV. The other two delivery techniques compared to the DCAT technique include 3-dimensional conformal radiotherapy (3DCRT) and intensity-modulated radiotherapy (IMRT), which use the same parameters for all three techniques.

**Results:**

The dosimetric parameters of the 3-group plans all met the RTOG 0813 protocol. Unsurprisingly, plan complexity parameters such as segments and MUs were significantly reduced in the DCAT plans by 159.56 and 925.90 compared to the IMRT plans, respectively (all *P* < 0.001). The delivery time of the DCAT plans was the least of 164.51 s (all *P* < 0.05). Compared to the IMRT plans, the γ-passing rates were higher in the DCAT plans (*P* < 0.001), with the most significant difference of 6.01% in the (2%, 1 mm) criteria. As for the interplay effect, the mean dose difference (MDD) in the DCAT plans was as good as the 3DCRT plans at different respiratory amplitudes but better than the IMRT plans (all *P* < 0.05), and the MDD of DCAT plans did not exceed 3% in all respiratory amplitude.

**Conclusion:**

In centers lacking the VMAT technique, implementing SBRT treatment based on the DCAT technique for inoperable early-stage centrally-located NSCLC patients with PTV volumes < 65 cc achieves better treatment efficiency and delivery accuracy while maintaining the plan quality.

## 1 Introduction

In recent years, the incidence of inoperable early-stage centrally-located non-small cell lung cancer (NSCLC) has been increasing with the increase in population aging and the availability of screening tools (1–3), and the stereotactic body radiotherapy (SBRT) is gradually becoming a standard of care (4–7). The characteristics of SBRT include fewer fractions, higher single doses, higher conformity, and accurate delivery (8). The tumors of early-stage centrally-located NSCLC are close to some critical organs, such as the ipsilateral proximal bronchus tree (PBT) and the healthy lungs, which may be damaged by the high dose of SBRT, thus failing to achieve optimal treatment. Therefore, SBRT planning for inoperable early-stage centrally-located NSCLC requires the most suitable delivery technique (4).

The most common delivery techniques for linac-based SBRT treatment include three-dimension conformal radiotherapy (3DCRT), intensity-modulated radiotherapy (IMRT), dynamic conformal arc therapy (DCAT), and volumetric-modulated arc therapy (VMAT) (9, 10). Many reports have confirmed that the VMAT technique has the highest comprehensive advantage. For example, Dwivedi et al. (11) found that VMAT-based SBRT plans for lung cancer were of better quality, with treatment time decreasing by 57.09% to 60.39% compared to 3DCRT. Xhaferllari et al. (12) concluded that VMAT had a dosimetric advantage compared to fixed-beam IMRT in SBRT treatment of early-stage NSCLC and significantly reduced treatment time. Rauschenbach et al. (13) thought that VMAT could lower the doses of all the organs at risk (OARs) compared to DCAT and 3DCRT. However, in the vast central and western regions of China, many condition-limited centers have only just completed the popularization of the fundamental three-dimension technique and lack the hardware and software to implement the VMAT technique, and the available SBRT techniques are 3DCRT, IMRT, and DCAT.

The 3DCRT technique has the most straightforward plan complexity of these three techniques and lacks the intensity modulation capability to protect adjacent OARs. In contrast, the IMRT technique is superior to the 3DCRT technique in almost all dosimetric parameters (14, 15). The DCAT technique is traditionally just the arc 3DCRT technique, which is not much different from the 3DCRT technique in OAR-sparing (16). However, the novel DCAT technique based on the Monaco treatment planning system (TPS) incorporates two new improvements, namely, variable dose rate (VDR) and segment shape optimization (SSO), which allow the linac to reduce the dose rate where a limited dose is needed, and allow the multi-leaf collimator (MLC) to move within a 5-mm range in and out of the targets, thus effectively improving the plan quality (17, 18). As a result, excellent dosimetric quality of the DCAT technique is demonstrated in treating small, rounded targets. Rauschenbach et al. (13) concluded that the DCAT technique was superior in high and low-dose spillage compared to the 3DCRT technique in the NSCLC-SBRT. Ming et al. (19) found that the mean heart dose of DCAT and IMRT were 2.3 Gy and 5.2 Gy in lung cancer radiotherapy, respectively, meaning that the DCAT technique was better than the IMRT in heart-sparing. Shi et al. (20) thought the DCAT technique was valuable and efficient for lung SBRT planning, and the plan quality met the RTOG protocols.

Several reports showed that the DCAT technique had advantages regarding plan complexity, delivery time and the γ-passing rates. Ong et al. (21) found that the MU efficiency (MU/Gy) was 187 ± 20, 179 ± 18, and 445 ± 84 for DCAT, 3DCRT and IMRT plans. Rauschenbach et al. (13) thought that when the single dose was normalized to deliver 20 Gy for comparison purposes, the mean MUs were 1880 ± 1260, 2540 ± 2020, and 3580 ± 1900, respectively, for DCAT, 3DCRT and VMAT. Moon et al. (22) concluded that the delivery time in liver SBRT for DCAT and VMAT were 3.6 ± 0.5 min and 4.5 ± 0.7 min, respectively. Lee et al. (23) considered that the γ-passing rate in lung SBRT for DCAT was 97.60 ± 2.41% under 2%/2mm criteria, which was high enough for accurate delivery.

An influencing factor that cannot be neglected during dose delivery in early-stage centrally-located NSCLC is respiratory motion, which may lead to deviations between the planned and delivered dose distributions in the form of dose blurring and interplay effects (24). Regardless of whether 3DCRT, IMRT or DCAT technique is used, dose blurring occurs, resulting in hot spots and cold spots in the target volume and an increasing dose to the adjacent OARs, so every effort must be made to minimize the interplay effects. Of these three techniques, the DCAT is less vulnerable to interplay effects. Netherton et al. (25) thought that the simpler the IMRT plan complexity, the lower the interplay effect. Burton et al. (26) found that the DCAT plans were sufficiently robust to overcome the interplay effect, which meant that the DCAT plans had a mean value of 6% dose deviation. However, the VMAT plans had a mean value of up to 21% dose deviation in single-fraction lung SBRT utilizing flattening filter-free (FFF) beams. Seco et al. (27) thought that daily intrafraction dose variation of more than 10% in IMRT plans was non-negligible and could potentially lead to biological effects.

Most of the literature has focused on only the dosimetric advantage of the DCAT technique, with few comprehensive analyses of its dosimetry, plan complexity, delivery time, the γ-passing rates and the interplay effect. This paper will comprehensively analyze the above factors to provide a reference for implementing SBRT using the DCAT technique in inoperable early-stage centrally-located NSCLC.

## 2 Methods

### 2.1 Patient cohort

Thirty-six consecutive patients with inoperable early-stage centrally-located NSCLC treated with SBRT were retrospectively selected after obtaining approval from the Ethics Committee of the Second Affiliated Hospital of Zhengzhou University (ethics number: 2023202). Due to its retrospective design, the Ethics Committee of the Second Affiliated Hospital of Zhengzhou University waived the need to obtain informed patient consent. All methods were carried out following relevant guidelines and regulations. The general conditions of the patients are shown in **Table 1**. Based on the RTOG 0236 and 0813 protocols (28, 29), the screened patients were required to fulfill the following criteria: planning target volume (PTV) no larger than 5 cm and located outside the 0.5 cm area and within the 2 cm area of the ipsilateral PBT or immediately adjacent to the mediastinal or pericardial pleura. Exclusion of patients with the tumors’ extent cannot be defined on CT (e.g., solid lesions around the tumor or lung atelectasis) and patients with ultracentral lung tumors.

**Table 1:** Summary of patients’ general conditions.

Item	Descriptions
Tumor Stage	14 cases of T1N0M0, 22 cases of T2N0M0
Age	32 to 73 years old, median age 51
Gender	19 males and 17 females
Tumor Location	13 cases in the lower lobe of the left lung, 14 cases in the lower lobe of the right lung and 9 cases in the middle lobe of the right lung
Target Size	ITV volume between 2.01 – 23.15 cc, average 16.64 cc; PTV volume between 5.82 – 63.78 cc, average 29.49 cc, max < 65 cc

ITV, internal target volume; PTV, planning target volume.

### 2.2 Positioning and contouring

Scanning was performed with 4DCT. Each patient underwent scanning on a 16-row big-bore CT scanner (Philips Medical Systems, Cleveland, OH) with a slice thickness of 3 mm.

All patients were placed in the supine position, with their arms holding a handle above their heads. The thoracic area was fixed using a thermoplastic film (Guangzhou Klarity Medical Equipment Co., Ltd., Guangzhou, China). The scanning range included all OARs to be evaluated. The recommended range was from the upper edge of the cricoid cartilage to the upper edge of the vertebral body of lumbar 2, with a minimum of 10 cm above the upper and lower boundaries of the tumors. The internal target volume (ITV) was delineated on the maximum intensity projection CT (MIPCT) by a radiation oncologist with expertise in lung SBRT. Based on the 4DCT, all patients had a respiratory amplitude of ≤ 5 mm in the 3D direction during free breathing. The PTV was created by adding an isotropic 5 mm margin to the ITV according to the RTOG recommendations (28, 30). The average intensity projection CT (AIPCT) was used for planning and dose calculations.

The OARs to be delineated included the ipsilateral lung and lung all (both excluding the ITV, total named as healthy lungs), the ipsilateral PBT, the spinal cord, the esophagus, the great vessels, the heart, the ipsilateral brachial plexus, and the skin.

### 2.3 Planning

The 3-group plans were redesigned for each patient in Monaco TPS (V6.0, Elekta Solution AB, Kungstensgatan 18, Stockholm, Sweden) using the XVMC (X-ray Voxelized Monte Carlo) algorithm (17). All the plans were delivered by an Infinity Linac equipped with an Agility collimator and a 6 MV X-ray beam (1400 MU/min). The prescription for all plans was 50 Gy/5 f.

In the 3DCRT plans, ten static, noncoplanar and nonopposing beams were used, and eight on the ipsilateral side at 30° intervals (table angle 0°) and two on the anterior side (table angle 90°), with the isocenter placed at the center of the PTV. Beam directions and weights were manually optimized according to the tumor location, mainly to achieve the mediastinal OAR-sparing. All beams in the 3DCRT plans used a collimation angle of 0°. The geometry for the IMRT plans was the same as for the 3DCRT plans. The IMRT delivery method was dynamic MLC, and the segment sequencing options were chosen as SSO and High Precision Leaf Position. The maximum number of segments per beam was 30, the minimum segment width was 0.70 cm, and the fluence smoothing option was medium and max. Sweep Efficiency and Allow Move Only Segments were selected. In the DCAT plans, two 210° arcs (table angle 0°) on the ipsilateral side, plus a 60° anterior arc (table angle 90°) were used, with the isocenter placed at the center of the PTV. The DCAT plans used different collimation angles between ±45° for each arc to minimize the tongue-and-groove effect (31). The angular increment was set to 10°, and SSO and VDR were selected. All plans’ dose grid and statistical uncertainty were 2.0 mm and 1%, respectively, and the Target Margin was selected as 0-1 mm.

In IMRT and DCAT planning, the dose constraints of the ipsilateral lung and the ipsilateral PBT should be met first and even prioritized over the PTV dose coverage. All plans were rescaled to fulfill 95% of the PTV covered by 100% prescription dose, and > 99% of the ITV covered by 100% prescription dose, and all hot spots (between 120% and 150% prescription dose) were located within the ITV. If mediastinal OARs overlap the PTV, the minimum dose for the PTV and ITV should be at least 70% and 90% of the prescription dose, respectively.

### 2.4 Dosimetric evaluation

The PTV was evaluated using D_98%_, D_2%_, homogeneity index (HI), conformity index (CI), and R_50%_, respectively, where D_98%_ and D_2%_ represent the PTV’s approximate minimum and maximum dose. Based on the RTOG 0813 protocol, D_2%_ decreases with decreasing PTV. At PTV = 70 cc, D_2%_ was < 86.0 Gy; at PTV = 50 cc, D_2%_ was <77.0 Gy; and at PTV < 10 cc, D_2%_ was < 57.0 Gy. For all PTV, D_98%_ was > 45.0 Gy. CI (32) and R_50%_ were used to describe the high and intermediate dose spillage for the 3-group plans. CI=PIV/TV, where PIV is the prescription isodose volume, and TV is the target volume. The value of CI is ≥1, where the closer the CI is to 1, the better. According to the RTOG 0813 protocol, CI < 1.5, preferably < 1.2. R_50%_ represents the 50% prescription isodose volume ratio to the PTV. Based on the RTOG 0813 protocol, R_50%_ increases as the PTV decreases. For example, R_50%_ is < 4.8 at PTV = 70 cc, R_50%_ is < 5.0 at PTV = 50 cc, and R_50%_ is < 7.5 at PTV <10 cc. HI describes the dose homogeneity degree within the PTV, HI = D_5%_/D_95%_ (33), where D_5%_ and D_95%_ represent the dose to cover 5% and 95% of the PTV, respectively. A lower HI is preferred, but pursuing a homogeneous dose within the PTV increases the OAR dose. Therefore, when the PTV does not contain organs that need to be preserved (34), the maximum HI should be set with caution. Based on the clinical practice in the department, HI < 1.6 is generally considered appropriate.

OARs were compared in compliance with the RTOG 0813 protocol and other relevant requirements in the literature (35, 36), including ipsilateral lung V_5Gy_ < 60% and V_20Gy_ < 20%, lung all V_5Gy_ < 40% and V_20Gy_ < 10%; ipsilateral PBT V_18Gy_ < 4 cc and D_max_ < 52.5 Gy; spinal cord V_22.5Gy_ < 0.25 cc, V_13.5Gy_ < 0.5 cc and D_max_< 32 Gy; esophagus V_27.5Gy_ < 5 cc and D_max_ < 52.5 Gy; heart V_32Gy_ < 15 cc and D_max_ < 52.5 Gy; great vessels V_47Gy_ < 10 cc and D_max_ < 52.5 Gy; ipsilateral brachial plexus V_30Gy_ < 3 cc and D_max_ < 32 Gy; skin V_30Gy_ < 10 cc and D_max_ < 32 Gy.

### 2.5 Plan complexity, delivery time, and the γ-passing rates

Plan complexity was weighted based on segments and MUs, as segments and MUs positively correlate with plan complexity (37). Delivery time was related to delivery efficiency, and they were recorded when measuring γ-passing rates using SRS MapCHECK (equipped with the StereoPHAN phantom) (Sun Nuclear, Melbourne, FL). Based on the AAPM TG-218 report and other documentation (38, 39), and taking into account the clinical practice in the department, the γ-passing rates were 2%/2mm > 95%, 2%/1mm > 85%, and 1%/2mm > 90%, while excluding data below 10% of the maximum dose.

### 2.6 Interplay effect

The interplay effects of the 3-group plans were all performed on a programmable dynamic respiratory phantom (CIRS 008A, Computer Imaging Reference Systems, Norfolk, USA). This phantom consists of a static chest model and a moving lung-equivalent rod with a spherical water-equivalent target that simulates a tumor’s motion. The study simulated the respiratory movements through a one-dimensional motion in the cranial-caudal direction.

A representative respiratory motion function was used to simulate the respiratory motion profile (40, 41). The motion function is defined as,


(1) $$\text{A}={\text{A}}_{0}\text{si}{\text{n}}^{6}\left(\text{πt}/\text{T}\right)$$

where t is the time, A_0_ is the respiratory amplitude, and T is the respiratory period. The respiratory movements of three respiratory amplitudes (20, 10 and 5 mm, peak-to-peak) were simulated for a 5 s respiratory period.

**Figure 1:**
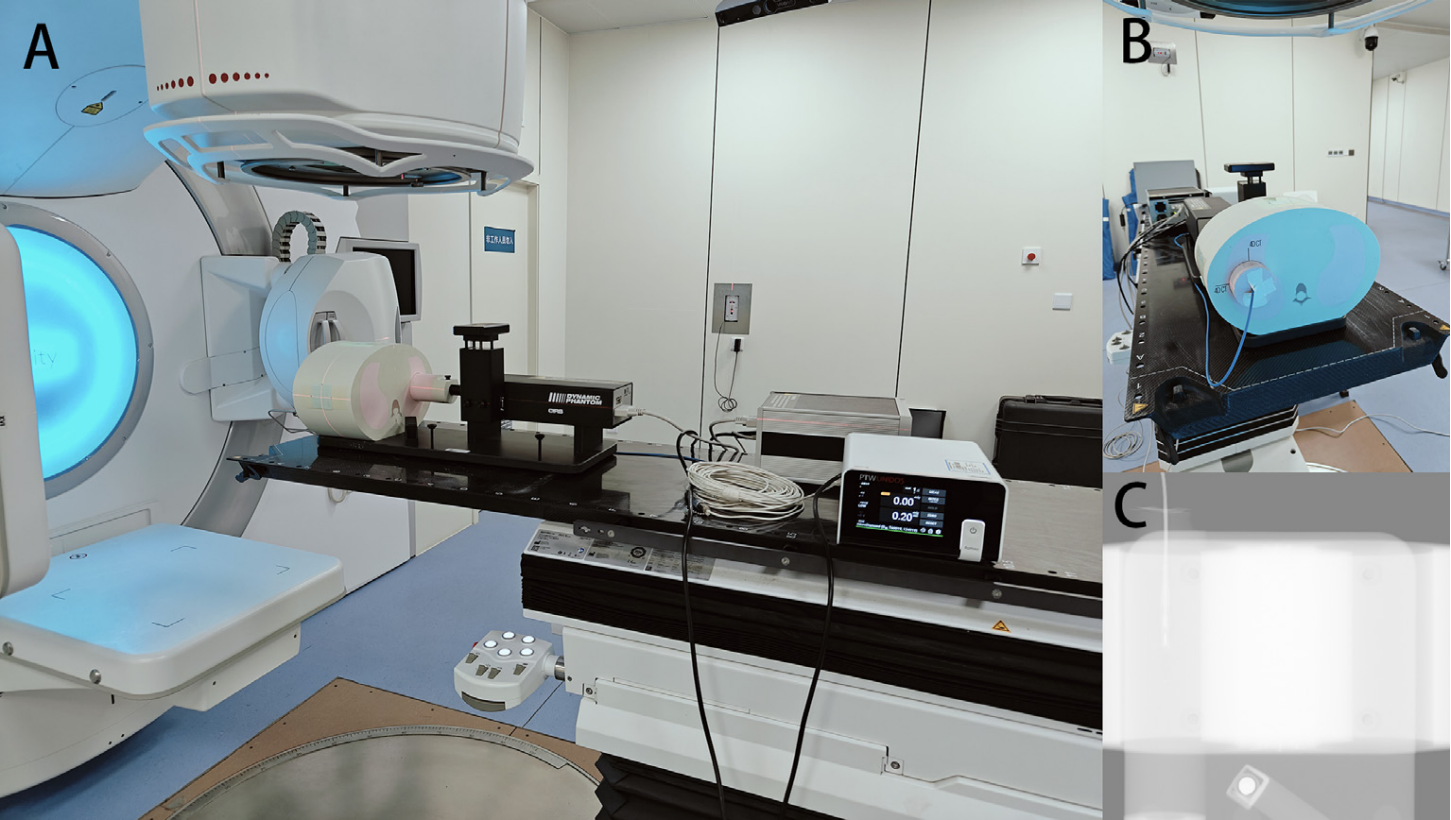
Schematic diagram of the combined use of the CIRS 008A phantom and the PTW microdiamond detector. **(A)** lateral view; **(B)** front view; **(C)** X-ray fluoroscopic view.

The point dose at the center of the target volume (phantom center) was measured using a center-located microdiamond detector (PTW, Freiburg, Germany) with a minimum sensitive volume of 0.004 mm^3^, as shown in **Figure 1**. The detector was cross-calibrated in water against a 0.6 cc farmer type chamber calibrated in a primary laboratory. Considering that the cable of the microdiamond detector is likely to generate artifacts on the CT of the phantom, and other tool will be used to measure the γ-passing rates of the plans, the phantom was used to directly measure the static point doses, and then measured the dynamic point doses under different respiratory amplitudes respectively. Starting from a random respiration phase, each of the collection was repeated three times per respiration amplitude for averaging, and the absolute value of the difference between the measured dynamic and static dose divided by the static dose was used to quantify the interplay effect, named as mean dose difference (MDD).

### 2.7 Statistical analysis

Data were analyzed using SPSS 25.0 (IBM SPSS Statistics for Windows, IBM Corp Version 25.0. Armonk, NY). The paired non-parametric Wilcoxon signed-rank test was used to compare any two plans. The confidence interval was 95%, with *P* < 0.05 indicating a statistically significant difference.

## 3 Results

### 3.1 Dosimetric parameters

**Figure 2:**
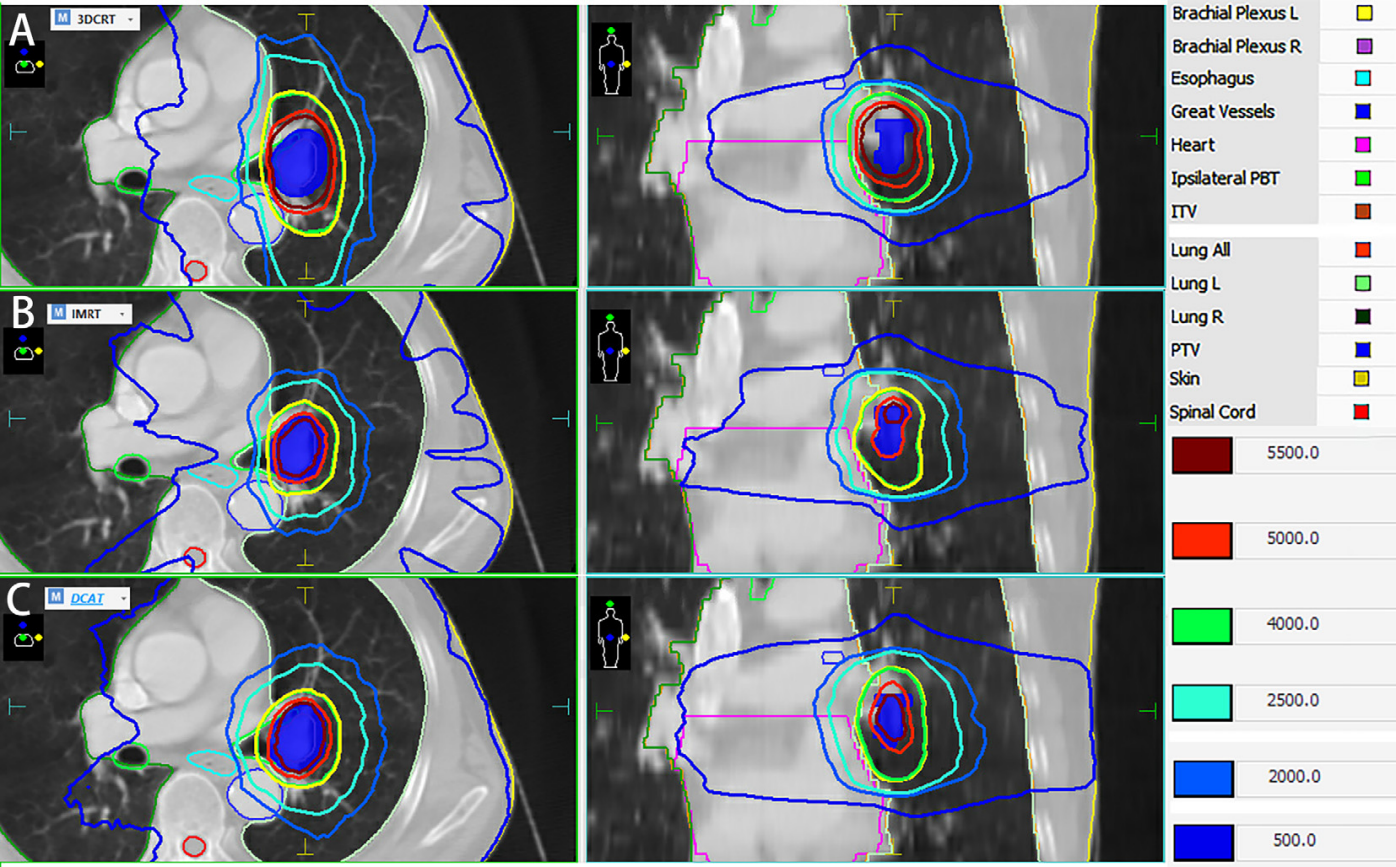
Dose distribution of the same case of 3DCRT (Group **A**), IMRT (Group **B**) and DCAT plans (Group **C**). The DCAT plan has a relatively uniform target dose distribution than the IMRT plan, and a tighter dose distribution than the 3DCRT plan.

**Figure 3:**
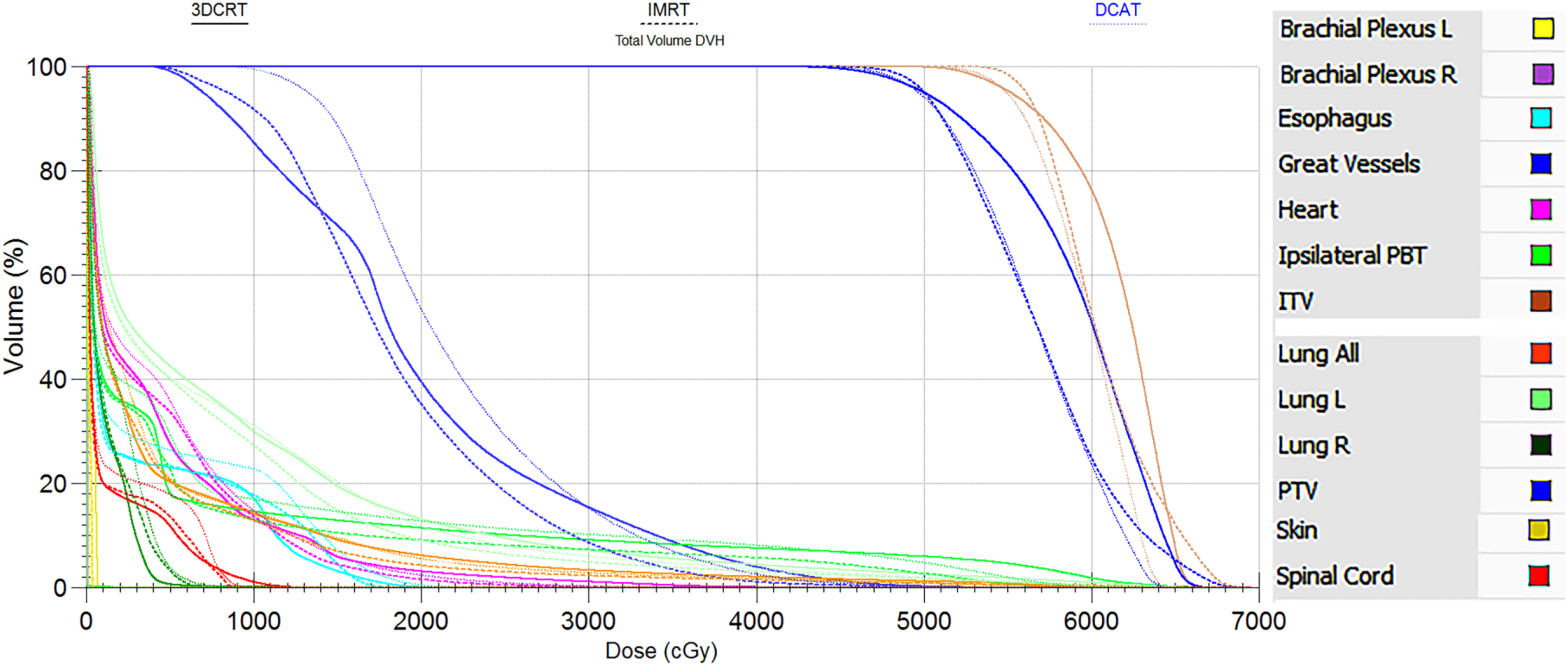
DVH comparison of the same case between 3DCRT (solid line), IMRT (long dash line) and DCAT plans (short dash line). The legend is displayed in the right side. DVH similarly supports the view that the DCAT plan has a relatively uniform target dose distribution than the IMRT plan, and a better OAR-sparing than the 3DCRT plan.

As can be seen in **Table 2**, the 3-group plans all met the RTOG 0813 protocol. The D_2%_, D_98%_, and HI of the 3-group plans were very similar, and the differences were not statistically significant (all *P* > 0.05). **Figures 2**, **3** show the transverse and coronal isodose lines and the DVH for the same case of the 3-group plans, and it can be seen that the DCAT plan had a more uniform target dose than the IMRT plan and a better OAR-sparing than the 3DCRT plan. The mean CI value of the DCAT plans was between IMRT plans and 3DCRT plans, and the differences were statistically significant (all *P* < 0.05). The mean R_50%_ of the DCAT plans was not as good as that of the IMRT plans but better than the 3DCRT plans, with a mean value of 5.01, and the differences were statistically significant (all *P* < 0.05).

**Table 2:** Summary of dosimetric parameters for the 3-group plans.

Parameters (mean [SD])		3DCRT (A)	IMRT (B)	DCAT (C)	*P* value		
					A VS. B	A VS. C	B VS. C
PTV	D_98%_(Gy)	44.86 (1.23)	43.59 (1.81)	44.02 (2.84)	0.953	0.886	0.518
	D_2%_(Gy)	61.80 (2.87)	66.69 (3.11)	64.55 (5.62)	0.051	0.083	0.178
	HI	1.29 (0.05)	1.27 (0.05)	1.28 (0.10)	0.127	0.072	0.913
	CI	1.36 (0.11)	1.11 (0.04)	1.15 (0.04)	0.008*	0.011*	0.005*
	R_50%_	5.64 (0.74)	4.36 (0.53)	5.01 (0.62)	<0.001*	0.015*	0.001*
Ipsilateral PBT	V_18Gy_ (%)	3.15 (1.12)	3.23 (1.12)	3.04 (1.06)	0.374	0.445	0.291
	D_max_ (Gy)	51.40 (2.99)	50.25 (3.41)	46.10 (3.74)	0.674	<0.001*	<0.001*
Ipsilateral lung	V_5Gy_ (%)	36.36 (7.33)	37.01 (7.00)	39.44 (7.99)	0.008*	<0.001*	<0.001*
	V_20Gy_ (%)	10.75 (3.43)	7.65 (2.68)	9.53 (3.61)	<0.001*	0.066	<0.001*
Lung all	V_5Gy_ (%)	18.60 (4.52)	20.55 (6.09)	21.71 (6.71)	0.006*	<0.001*	<0.001*
	V_20Gy_ (%)	5.80 (1.43)	3.71 (0.27)	4.59 (1.88)	<0.001*	0.005*	<0.001*
Spinal cord	D_max_ (Gy)	11.95 (2.57)	9.74 (2.70)	10.11 (5.36)	0.048*	0.260	0.648
Esophagus	D_max_ (Gy)	21.37 (6.98)	20.70 (10.66)	20.55 (11.63)	0.173	0.069	0.850
Heart	V_32Gy_ (%)	2.50 (3.16)	1.31 (2.04)	1.12 (1.85)	0.028*	<0.001*	0.465
	D_max_ (Gy)	39.55 (13.39)	35.97 (15.76)	34.45 (15.20)	<0.001*	0.008*	0.018*
Great vessels	V_47Gy_ (%)	0.20 (0.28)	0.09 (0.18)	0.16 (0.39)	0.310	0.635	0.199
	D_max_ (Gy)	48.52 (4.82)	42.51 (9.93)	40.57 (10.87)	0.263	0.031*	0.118
Ipsilateral brachial plexus	D_max_ (Gy)	0.33 (0.30)	0.32 (0.15)	0.28 (0.25)	0.377	0.112	0.385
Skin	D_max_ (Gy)	10.53 (1.04)	14.60 (1.15)	9.74 (1.99)	0.008*	0.224	<0.001*

SD, standard deviation; 3DCRT, 3-dimensional conformal radiotherapy; IMRT, intensity-modulated radiotherapy; DCAT, dynamic conformal arc therapy; VS., versus; PTV, planning target volume; D_98%_, dose to 98% of the target volume; D_2%_, dose to 2% of the target volume; HI, homogeneity index; CI, conformity index; R_50%_, the ratio of the 50% prescription isodose volume to the PTV; PBT, proximal bronchial tree; V_x Gy_, the ratio of the volume received > x Gy dose by an organ to the total volume; D_max_, the maximum point dose to an organ. A statistically significant difference result is indicated by an asterisk (*).

The DCAT plans were superior in the ipsilateral PBT-sparing among the 3-group plans, especially in the D_max_ parameter (all *P* < 0.001). The evaluation parameters of the healthy lungs in the DCAT plans were all inferior to those in the IMRT plans but were superior to those in the 3DCRT plans (Lung All V_20Gy_). However, the most significant difference in mean value among the 3-group plans was only 7.81% higher (DCAT vs. 3DCRT, ipsilateral lung V_5Gy_). For the remaining OARs listed in **Table 2**, the corresponding parameters of the DCAT plans were mostly between 3DCRT and IMRT plans, and the 3-group plans were all above the qualified line. Some of the OARs’ data were not presented in **Table 2** because all the values among the 3-group plans were 0 (spinal cord V_22.5Gy_, ipsilateral brachial plexus V_30Gy_, and skin V_30Gy_), or the majority of them were 0 (spinal cord V_13.5Gy_, esophagus V_27.5Gy_).

### 3.2 Plan complexity, delivery time, the γ-passing rates, and the interplay effect

Regarding the plan complexity, the DCAT plans showed a significant reduction in segments and MUs compared to the IMRT plans, mean reduction in segments by 159.56 and MUs by 925.90 *(all P* < 0.001), as shown in **Table 3**. The mean delivery time of the DCAT plans was the least of 164.51 s (all *P* < 0.05). The γ-passing rates of 3-group plans were qualified under different criteria (2%/2mm, 1%/2mm, and 2%/1mm), as shown in **Table 3**. However, the γ-passing rates of the DCAT plans were higher than those of the IMRT plans under all criteria (*P* < 0.001), and the advantage became more and more evident as the criteria became more and more stringent. For example, under the criteria of (2%/1mm), the γ-passing rates of the DCAT plans got a mean improvement of 6.01% compared to the IMRT plans (*P* < 0.001). As for the interplay effect, with the respiratory amplitude decreased, the MDD decreased in all the 3-group plans. At different respiratory amplitudes, the MDD in the DCAT plans was as good as the 3DCRT plans but better than the IMRT plans (all *P* < 0.05). The MDD of DCAT plans did not exceed 3% at expected respiratory amplitudes.

**Table 3:** Summary of other parameters for the 3-group plans.

Parameters (mean [SD])		3DCRT (A)	IMRT (B)	DCAT (C)	*P* value		
					A VS. B	A VS. C	B VS. C
Segments	N/A	10	261.68 (11.10)	102.12 (27.25)	N/A	N/A	<0.001*
MUs	N/A	1648.93 (76.43)	2781.36 (451.68)	1855.46 (269.34)	0.008*	0.842	<0.001*
Delivery time (s)	N/A	192.32 (18.54)	253.47 (23.97)	164.51 (15.07)	<0.001*	0.017*	<0.001*
γ-passing rates (%)	(2%/2mm)	99.27 (0.45)	96.34 (1.98)	98.99(1.11)	<0.001*	0.674	<0.001*
	(1%/2mm)	97.16 (1.87)	93.61 (2.78)	96.85(1.97)	<0.001*	0.138	<0.001*
	(2%/1mm)	93.55 (2.98)	87.13 (3.77)	93.14 (2.91)	<0.001*	0.164	<0.001*
MDD (%)	20mm	2.05 (1.62)	4.39 (5.47)	2.84 (2.48)	<0.001*	0.321	0.035*
	10mm	1.68 (0.56)	3.73 (4.68)	1.89 (1.45)	<0.001*	0.647	0.006*
	5mm	0.78 (0.22)	2.35 (1.49)	1.08 (0.14)	<0.001*	0.257	0.014*

SD, standard deviation; 3DCRT, 3-dimensional conformal radiotherapy; IMRT, intensity-modulated radiotherapy; DCAT, dynamic conformal arc therapy; VS., versus; MUs, monitor units; N/A, not applicable; MDD, mean dose difference. A statistically significant difference result is indicated by an asterisk (*).

## 4 Discussion

In this paper, we compared the dosimetric parameters, plan complexity, delivery time, the γ-passing rates, and the interplay effect of the 3DCRT, the DCAT, and the IMRT plans for 36 patients with inoperable early-stage centrally-located NSCLC (PTV < 65 cc), and analyzed the feasibility of the DCAT plans to implement SBRT treatment (50Gy/5fx). The results showed that all dosimetric parameters of the 3-group plans met the RTOG 0813 protocol, and the DCAT plans showed the highest comprehensive advantage.

As shown in **Table 2** and **Figure 2**, the D_2%_, D_98%_, and HI of the DCAT plans were very similar to the other two plans (all *P* > 0.05), and the DCAT plans had moderate CI and R_50%_ values among the 3-group plans. The above results are similar to the findings of several authors. For example, Goto et al. (16) found that the DCAT plans (median 1.3) had better CI than the 3DCRT plans (median 2.2), which was similar to the results of Peterlin et al. (42). Yau et al. (43) thought that the DCAT plans could offer similar dosimetric benefit compared to the IMRT plans. It is reported that the clinical outcomes of the DCAT plans were pretty good, and the 1-year local control of DCAT-SBRT was 96.7%, 1-year overall survival was 93.1% in early-stage lung cancer (44).

The most common side effects of SBRT treatment for centrally-located lung cancer include fatal hemoptysis (45) and fatal radiation pneumonitis in patients with poor respiratory function (46–48), particularly those with underlying interstitial lung disease. Therefore, the dose to the ipsilateral PBT and healthy lungs must be strictly controlled according to the RTOG 0813 protocol. As shown in **Table 2**, the DCAT plans were superior in protecting the ipsilateral PBT in parameters of D_max_ (all *P* < 0.001). The lung evaluation parameters in the 3-group plans were substantially lower than the RTOG 0813 protocol, and the DCAT plans got relatively lower V_20Gy_. It has been reported that only 4.6% of symptomatic grade 2 radiation pneumonitis in similar lung evaluation parameters in the DCAT plans (44). In addition, **Table 2** showed the DCAT plans had advantages in most remain OARs. In summary, all three techniques produced dosimetrically qualified plans much smaller in absolute values than those required by the RTOG0813 protocol. Therefore, no significant differences in radiotoxicity were expected.

In addition to the dosimetric advantage, the most significant advantages of the DCAT plans are suitable plan complexity, delivery time, γ-passing rates, and the interplay effect (49–52). As can be seen in **Table 3**, the DCAT plans required fewer segments and MUs to deliver the same dose compared to the IMRT plans (13), with an average reduction in segments and MUs of 159.56 and 925.90, respectively (all *P* < 0.001). As a result, the mean delivery time of the DCAT plans was the least of 164.51 s (all *P* < 0.05), which could improve the patient’s comfort, especially in deep inspiration breath-hold radiotherapy (53). Another benefit of fewer segments and MUs may be the higher γ-passing rates compared to the IMRT plans (54). The results in **Table 3** showed that the γ-passing rates in the DCAT plans were more significant than that in the IMRT plans under all criteria (*P* < 0.001), and the advantage became more and more evident as the criteria became more and more stringent. This is due to the rounded segment shape and large segment area of the DCAT plans (17, 18, 44, 53, 55). **Table 3** also showed that the DCAT plans had a relatively lower interplay effect among the 3-group plans. At expected respiratory amplitudes (5-20 mm), the MDD did not exceed 3%. The interplay effect can lead to hot and cold spots within the target (40), reducing the tumor control probability. The study showed that the interplay effect of the DCAT plans was significantly smaller than that of the IMRT plans but similar to the 3DCRT plans. Considering the MLC leaves in the DCAT plans moved around the edge of the moving target, the findings of this study were consistent with the study of Ge et al. (50). This is especially critical in the SBRT treatment of NSCLC with respiratory motion. After all, the interplay effect is one of the biggest concerns in conducting chest SBRT treatment in the free-breathing condition (56). Therefore, the DCAT technique may be the best choice in some condition-limited centers when implementing inoperable early-stage centrally-located NSCLC SBRT (57).

Implementing the DCAT plans in this paper is based on two premises. The first point is that the targets must be far from the OARs (58), especially the ipsilateral PBT because the DCAT plans have low dose modulation and are less capable of OAR-sparing. The second point is that the maximum PTV volume included in this paper is < 65 cc, so no analysis is given for a larger PTV volume. One of the shortcomings of this paper is that it does not involve the research on multiple lesions. Therefore, we look forward to conducting in-depth research on radiotherapy techniques for multiple lesions in the upcoming experiments.

## 5 Conclusions

In centers lacking the VMAT technique, the study herein supports the DCAT technique as the first choice for SBRT treatment of inoperable early-stage centrally-located NSCLC (PTV < 65cc) because of certain advantages in terms of adequate OAR-sparing, less treatment time, high γ-passing rates, and low interplay effect.

## Data Availability

The original contributions presented in the study are included in the article/supplementary material. Further inquiries can be directed to the corresponding author.
